# Integrated transcriptome and metabolome analysis reveals that flavonoids function in wheat resistance to powdery mildew

**DOI:** 10.3389/fpls.2023.1125194

**Published:** 2023-02-01

**Authors:** Wenjing Xu, Xiaoyi Xu, Ran Han, Xiaolu Wang, Kai Wang, Guang Qi, Pengtao Ma, Takao Komatsuda, Cheng Liu

**Affiliations:** ^1^ Crop Research Institute, Shandong Academy of Agricultural Sciences, Jinan, Shandong, China; ^2^ National Engineering Laboratory of Wheat and Maize, Jinan, Shandong, China; ^3^ Key Laboratory of Wheat Biology and Genetic Improvement in the North Huang and Huai River Valley of Ministry of Agriculture, Jinan, Shandong, China; ^4^ Shandong Wheat Technology Innovation Center, Jinan, Shandong, China; ^5^ School of Life Sciences, Yantai University, Yantai, Shandong, China

**Keywords:** wheat, powdery mildew, transcriptome, metabolome, flavonoids

## Abstract

Powdery mildew is a fungal disease devastating to wheat, causing significant quality and yield loss. Flavonoids are important secondary plant metabolites that confer resistance to biotic and abiotic stress. However, whether they play a role in powdery mildew resistance in wheat has yet to be explored. In the present study, we combined transcriptome and metabolome analyses to compare differentially expressed genes (DEGs) and differentially accumulated flavonoids identified in plants with and without powdery mildew inoculation. Transcriptome analysis identified 4,329 DEGs in susceptible wheat cv. Jimai229, and 8,493 in resistant cv. HHG46. The DEGs were functionally enriched using Gene Ontology and Kyoto Encyclopedia of Genes and Genomes, revealing the flavonoid synthesis pathway as the most significant in both cultivars. This was consistent with the upregulation of flavonoid synthesis pathway genes observed by quantitative PCR. Metabolome analysis indicated flavone and flavonol biosynthesis pathways as the most significantly enriched following powdery mildew inoculation. An accumulation of total flavonoids content was also found to be induced by powdery mildew infection. Exogenous flavonoids treatment of inoculated plants led to less severe infection, with fewer and smaller powdery mildew spots on the wheat leaves. This reduction is speculated to be regulated through malondialdehyde content and the activities of peroxidase and catalase. Our study provides a fundamental theory for further exploration of the potential of flavonoids as biological prevention and control agents against powdery mildew in wheat.

## Introduction

Wheat – a major food staple worldwide – is threatened by a number of diseases, including powdery mildew, caused by the fungus *Blumeria graminis* (Singh et al., 2016; Savary et al., 2019). This condition damages and affects the growth of the leaf blade, sheath, glume, and awn, presenting as white spots and resulting in a ~50% yield reduction (Livak and Schmittgen, 2001; Conner et al., 2003; Wang et al., 2005). A typical method of disease management is through chemical control, which is effective but costly and polluting to the environment (Bennett, 1984; Bliffeld et al., 1999). Hence, identifying genes involved in the resistance to this infection and applying them to breed-resistant cultivars is a cheaper and more sustainable alternative for controlling the disease (Koller et al., 2018; Sun et al., 2018). To date, more than 240 powdery mildew-resistant genes in wheat or wheat relatives have been identified; however, few of these have been utilized in the enhancement of wheat crops, either due to loss of resistance to powdery mildew (Limpert et al., 1987; Duan et al., 1998; Qiu & Zhang, 2004; Wang et al., 2005) or the carrier lines have agricultural disadvantages (Li et al., 2007). These effects can be balanced by developing a biological control to prevent this disease in an effective and environmentally friendly manner.

Flavonoids, a widespread class of secondary metabolites in plants, can be classified into 12 subgroups according to the degree of oxidation of the heterocyclic ring and the number of hydroxyl or methyl groups on the benzene ring (Sasaki & Nakayama, 2015): chalcones, stilbenes, aurones, flavanones, flavones, isoflavones, phlobaphenes, dihydroflavonols, flavonols, leucoanthocyanidins, proanthocyanidins, and anthocyanins. These metabolites are normally generated from 4-coumaroyl-CoA, and primarily catalyzed by the enzymes chalcone synthase (CHS), chalcone isomerase (CHI), dihydroflavonol 4-reductase (DFR), flavonol synthase (FLS), flavone synthase (FNS), and anthocyanidin synthase (ANS) (Lewis et al., 2011). Flavonoids play a number of important roles in plants, not only in growth and developmental processes, but also by functioning as antioxidants under conditions of biotic and abiotic stress (Taylor & Grotewold, 2005). As one of the most active groups of antioxidants in plant resistance to stress, flavonoids can regulate factors of the redox system to activate the plant immune system, triggering the production of enzymes such as peroxidase (POD), superoxide dismutase (SOD), and catalase (CAT), which act in plant resistance to diseases (Cavaiuolo et al., 2013). It has been reported that flavonoids can prevent the occurrence of powdery mildew in commercial cucumber and strawberries (Fofana et al., 2005; Bajpai et al., 2019). However, whether they act against powdery mildew in wheat has not yet been investigated to the best of our knowledge.

Multi-omics techniques, including genomics, transcriptomics, proteomics, and metabolomics, can be used to explore the differences in plants with and without powdery mildew treatment. Integrated transcriptome and metabolome analysis is a powerful tool to investigate the mechanism of resistance to powdery mildew. This method was used to study resistance responses in Tibetan hull-less barley (Yuan et al., 2018), to assess transcriptional and metabolic changes in susceptible infected grapes (Pimentel et al., 2021), and to illustrate the key components of the defense pathways in the resistance of the *Arabidopsis mlo2mlo6mlo12* triple mutant (Kuhn et al., 2017). However, integrating transcriptomics and metabolomics to analyze the mechanisms of flavonoids’ resistance to powdery mildew in wheat has not been reported to date.

In the present study, we analyzed the role of flavonoids in wheat resistance to powdery mildew using integrated transcriptome and metabolome analysis. Differentially expressed genes (DEGs) and differentially accumulated flavonoids (DAFs) in susceptible and resistant wheat cultivars were studied to identify the main pathways and flavonoids that were affected following powdery mildew inoculation. The results of the transcriptome and metabolome analyses were validated by quantitative PCR (qPCR) and flavonoids content measurements. Furthermore, malondialdehyde (MDA) content and POD, SOD, and CAT activities were evaluated to illustrate the function of flavonoids in the resistance of wheat to powdery mildew.

## Materials and methods

### Plant materials

The powdery mildew-susceptible wheat cultivar Jimai229 was bred and kept at the Crop Research Institute (Shandong Academy of Agricultural Science, Jinan, China). The powdery mildew-resistant strain (acc. no. HHG46; possessing gene *Pm12*) was provided by Professor Huagang He, Jiangsu University, China.

### RNA extraction and RNA-seq analysis

Transcriptome analyses of the susceptible cv. Jimai229 and resistant cv. HHG46 were conducted seven days following powdery mildew inoculation alongside control plants without inoculation. Three biological replicates were performed for each treatment. Each replicate contained mixed leaf samples from at least five different plants. All samples were immediately frozen in liquid nitrogen and stored at -80°C.

Total RNA was extracted from wheat leaves using TRIzol reagent (Sangon Biotech Co., Ltd., Shanghai, China) according to the manufacturer’s protocols, and the RNA samples were sent for transcriptome analysis to Novogene Co., Ltd. (Beijing, China). The RNA-seq library was constructed using the NEBNext^®^ Ultra™ Directional RNA Library Prep kit (New England Biolabs, Ipswich, USA) and sequenced using the NovaSeq 6000 system (Illumina, Inc., San Diego, CA). Raw reads were obtained using illumine NovaSeq 6000 (illumine, USA) and analyzed for quality using fastp (v0.19.7;fastp -g -q 5 -u 50 -n 15 -l 150) (Chen et al., 2018). Unique reads were mapped to the wheat genome IWGSC RefSeq v2.0 assembly (acc. no. GCA_900519105) using HISAT2 v2.0.5 (Kim et al., 2019). The fragments per kilobase per transcript per million mapped reads (FPKM) were calculated as described by Li (Li et al., 2010), and the number of reads mapped to each gene were counted with featureCounts v1.5.0-p3 (http://subread.sourceforge.net). The FPKM for each gene was then calculated based on the length of the gene and read count mapped to it. A differential expression analysis of both susceptible and resistant cultivars with and without powdery mildew infection was performed using the DESeq2 v1.20.0 and edgeR v3.22.5 R packages (both https://bioconductor.org/packages/3.16/bioc/). The resulting P-values were adjusted using the Benjamini-Hochberg approach for controlling the false discovery rate. Genes with an adjusted P ≤ 0.05, as found by DESeq2, were assigned as differentially expressed.

DEG analysis was carried out in two groups: JM229-pm/JM229-0 and HHG46-pm/HHG46-0 (JM229, Jimai229; -pm, with powdery mildew treatment; -0, without powdery mildew treatment). To infer the putative functions of the DEGs, Gene Ontology (GO) and Kyoto Encyclopedia of Genes and Genomes (KEGG) enrichment analyses were conducted. GO analysis was implemented using the clusterProfiler R package v3.8.1 (https://bioconductor.org/packages/release/bioc/html/clusterProfiler.html), in which gene length bias was corrected. GO terms with corrected P<0.05 were considered significantly enriched. The clusterProfiler package was also used to test the statistical enrichment of the DEGs in KEGG pathways.

### Flavonoids-targeted metabolome analysis

The samples that were analyzed for their transcriptome were also used for metabolome analysis, which was carried out by MetWare Biotechnology Co., Ltd. (Wuhan, China). Each fresh leaf sample (0.6 g) was freeze-dried in a vacuum freeze-dryer (SCIENTZ-100F; Scientz Biotechnology Co., Ltd., Ningbo, China) and ground using a mixer mill (MM 400; Retsch GmbH, Haan, Germany) with a zirconia bead for 1.5 min at 30 Hz. The 50 mg lyophilized samples were each dissolved in 1.2 ml 70% methanol. Following centrifugation at 13,400 g for 3 min, the extracts were filtered through a SCAA-1040.22-μm pore-size filter (Anpel Laboratory Technologies, Inc., Shanghai, China) and analyzed by means of UPLC-ESI-MS/MS (UPLC: ExionLC™ ADsystem; MS: QTRAP 4500; both AB Sciex Pte. Ltd., Singapore).

Differentially accumulated metabolites were determined based on the cut-off values of variable importance for projection ≥1 and absolute Log2FC≥1.0. Identified metabolites were annotated according to the KEGG COMPOUND database (http://www.genome.jp/kegg/compound/), and then mapped using the KEGG PATHWAY database (http://www.genome.jp/kegg/pathway.html) to determine the metabolic pathways that were most strongly associated with resistance to powdery mildew.

### Correlation analysis of the transcriptome and metabolome datasets

A joint analysis of the DEGs and DAFs was carried out to determine the degree of enrichment of the pathways through a correlation heat map, correlation matrix, and association network diagram. The pathway information that was in common between the DEGs and DAFs was mapped to KEGG. Pearson correlation coefficients were calculated for the metabolome and transcriptome data integration. Log conversion of the data was performed uniformly before analysis; the data were analyzed using the cor function in R (version 3.5.1), and the screening criterion was a Pearson correlation coefficient >0.8.

### Gene expression assay by qPCR

qPCR was performed to confirm the results of the transcriptome analysis. Total RNA (500 ng), extracted as described above, was reverse-transcribed to first-strand cDNA using a commercial kit (Evo M-MLV Mix kit with gDNA Clean for qPCR; item no. AG11728; Accurate Biotechnology (Hunan), Changsha, China). Gene-specific forward and reverse PCR primers targeting *CHS*, *CHI*, *DFR*, *FNS*, *FLS*, and *ANS* were designed based on their cDNA sequences using Primer Premier 5 software (listed in **Table S1**) and manufactured by Tsingke Biotechnology Co., Ltd. (Beijing, China). qPCR was performed using the SYBR^®^ Green Pro Taq HS Premixed qPCR kit (item no. AG11701, Accurate Biotechnology (Hunan), Changsha, China) and the Cycler QTM real-time PCR detection system (Bio-Rad, Hercules, CA, USA). Each 20 μl reaction consisted of 10 μl SYBR^®^ Green PCR Master Mix, 2 μl diluted cDNA, and 0.1 μM forward and reserve primers. The cycling conditions were as follows: 95°C for 5 min, followed by 40 cycles of denaturation at 95°C for 10 s and annealing at 60°C for 30 s. The wheat actin gene was used as an internal control. The mRNA was quantified according to the 2^–ΔΔC^_T_ method (Livak & Schmittgen, 2001).

### Measurement of total flavonoids content

Seedlings were collected from wheats cv. Jimai229 and HHG46 seven days after inoculation with powdery mildew, as well as from control plants without inoculation. Three biological replicates were performed for each treatment. Each replicate contained mixed leaf samples from at least five different plants. The total flavonoids content of the wheat leaves was measured using the Total Flavonoids kit (ADS-F-KY007-48; AIDISHENG Biotechnology Co., Ltd., Jiangsu, China), according to the manufacturer’s protocol.

### Flavonoids treatment and evaluation of resistance to powdery mildew

Seeds of wheat cv. Jimai29 were sown in 20 x15 cm flowerpots (15 seeds per pot). Three experimental groups were set up: mock treatment, PM (powdery mildew inoculation, a mix of powdery mildew from Shandong Province), and PM+F (powdery mildew inoculation and flavonoids treatment), with 5 pots per treatment in one square-glass cover. For the flavonoids treatment, flavonoids were dissolved to a 10 mM concentration in 100% ethanol and diluted with distilled water to make the final 100 μM solution.

The wheat seeds in flowerpots were kept in a greenhouse under conditions of a 16/8h day/night photoperiod, a temperature of 22 ± 2°C, and 97–99% air humidity for five days before treatment. For the mock group, the plants were watered with autoclave water. In the PM and PM+F groups, the plants were inoculated with the powdery mildew mixture in the first leaf of each seedling. On days one, three, and five after the inoculation, the plants of the PM+F group were watered with the flavonoids solution (100 μM; 20 ml per pot) and the other groups were watered with 20 ml autoclaved water per pot. Images were captured of the three groups to record any differences in phenotype six days after the inoculation. The infection types of the plants were classified as 0–4, as described previously (Gong et al., 2017), where 0=immune; 0;= almost immune; 1=highly resistant; 2=moderately resistant; 3=moderately susceptible; and 4=highly susceptible. Classes 0–2 were regarded as resistant, while 3–4 were considered susceptible to infection.

### Measurement of MDA content and SOD, POD, and CAT activity

Samples were taken from cv. Jimai229 and HHG46 plants cultivated under the same conditions as described above. Three biological replicates were used for each treatment. Each replicate contained mixed leaf samples from at least five different plants. MDA content and the activity of SOD, POD, and CAT in the wheat leaves were measured using commercially available kits (cat. nos. ADS-F-YH002, ADS-F-KY001, ADS-F-KY003 and ADS-F-KY002, respectively; all AIDISHENG Biotechnology Co., Ltd., Jiangsu, China).

### Statistical analysis

Each experiment was set up with three biological replicates, and all data were expressed as mean ± standard deviation. Differences between groups were determined using one-way ANOVA (t-test). The data were analyzed using GraphPad Prism software 9.0.

## Results

### DEG analysis

Following cleaning and quality control of the RNA-seq results, approximately132.37 Gb were obtained, with an average of 11.03 Gb per sample. The average Q30 value (percentage of bases with a sequencing error rate of <0.1%) was 93.62% and GC content ranged between 52.72 and 58.47% (**Table S2**). The correlation coefficients between the groups were in the range of 0.457 to 0.958 (**Table S3**). These results indicated that the sequencing quality was reliable and suitable for further analysis.

**Figure 1:**
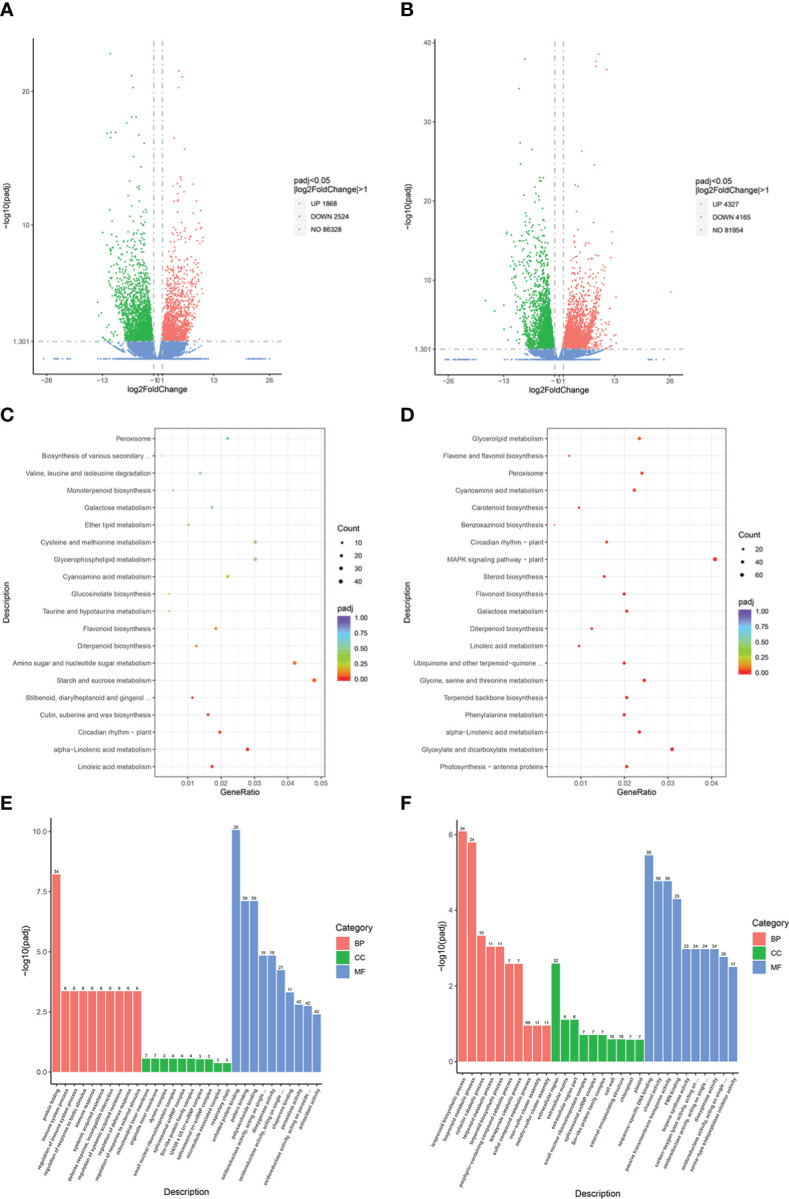
DEG analyses of wheat cv. Jimai229 and HHG46 with vs without powdery mildew inoculation. Volcano plots indicating DEGs in **(A)** JM229-pm/JM229-0 and **(B)** HHG46-pm/HHG46-0. Kyoto Encyclopedia of Genes and Genomes enrichment analysis of the DEGs from **(C)** JM229-pm/JM229-0 and **(D)** HHG46-pm/HHG46-0. Gene Ontology enrichment analysis of the DEGs from **(E)** JM229-pm/JM229-0 and **(F)** HHG46-pm/HHG46-0. BP, biological process; CC, cellular component; DEG, differentially expressed gene; MF, molecular function.

A total of 12,884 genes were obtained from the RNA-seq data. DEGs were identified based on an absolute log_2_fold-change ≥1 with a false discovery rate of ≤ 0.05, and were mapped against the GO and KEGG public databases to determine their potential functions. Volcano plots revealed 4,392 DEGs in the JM229-pm/JM229-0 set (2,524 downregulated; 1,868 upregulated genes), while 8,492 DEGs were identified in the HHG46-pm/HHG46-0 set (4,165 downregulated; 4,327 upregulated) (**Figures 1A, B**). A total of 109 terms were enriched in the KEGG analysis of the Jimai229 DEGs, including ‘linoleic acid metabolism’, ‘alpha-linolenic acid metabolism’, ‘circadian rhythm – plant’, ‘cutin, suberine, and wax biosynthesis’, and ‘flavonoids biosynthesis’ (**Figure 1C**). Among the 118 enriched terms from the HHG46 DEGs, ‘flavone and flavonol biosynthesis’ was enriched in addition to those identified in Jimai229, indicating that the production of flavone and flavonol may be related to powdery mildew resistance (**Figure 1D**). The GO analyses demonstrated that immune response, the membrane system, and oxidoreductase activity were induced by powdery mildew in Jimai229, and that the isoprenoid biosynthetic and metabolic processes were additionally involved in the HHG46 strain (**Figures 1E, F**). In order to compare the transcription levels of flavonoids-related genes between the mock and powdery mildew treatment, 20 genes from the JM229-pm/JM229-0 set and 48 genes from theHHG46-pm/HHG46-0 set that were potentially associated with flavone and flavonol biosynthesis were selected (**Table S4**). Of these, 6 were upregulated following inoculation with powdery mildew in Jimai229 and 12 inHHG46, including flavonoids O-methyltransferase-like protein, flavonoids 7-sulfotransferase 3a, flavonoids 3’-monooxygenase, UDP-glucose flavonoids 3-O-glucosyltransferase 7, anthocyanidin 3-O-glucosyltransferase, dimethylnonatriene synthase, flavonoids 3’-monooxygenase, and flavonoids 3’,5’-hydroxylase, suggesting that these genes play important roles in wheat resistance to powdery mildew.

### Flavonoids-targeted metabolome analysis

**Figure 2:**
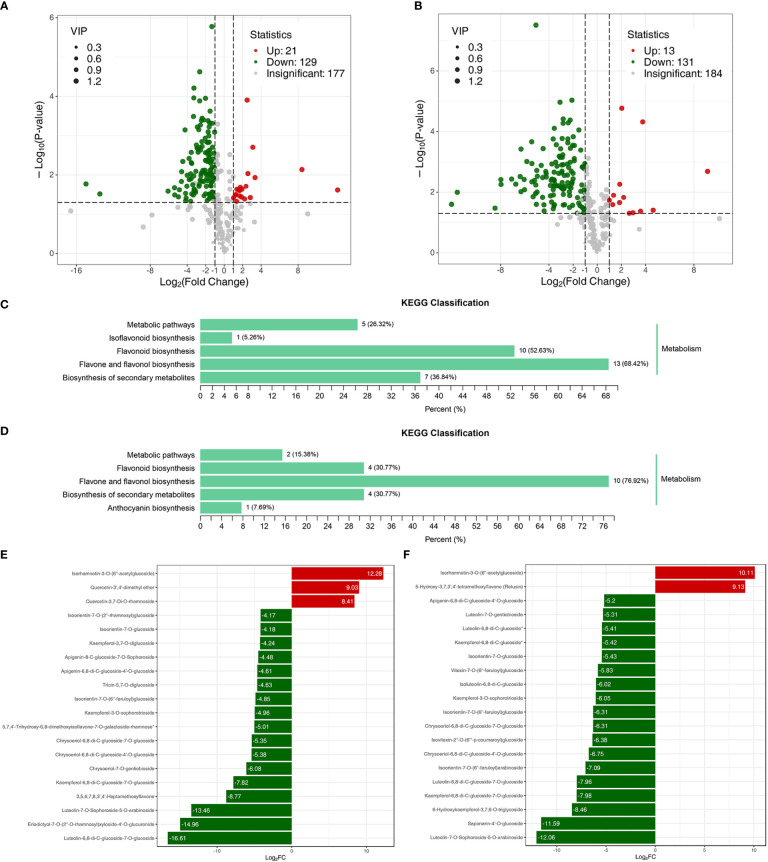
DAF analyses of wheat cv. Jimai229 and HHG46 with vs without powdery mildew inoculation. Volcano plots indicating DAFs in **(A)** JM229-0/JM229-pm and **(B)** HHG46-0/HHG46-pm. KEGG classification analysis of the DAFs from **(C)** JM229-0/JM229-pm and **(D)** HHG46-0/HHG46-pm. The top 20 DAFs from **(E)** JM229-0/JM229-pm and **(F)** HHG46-0/HHG46-pm. Red, upregulated DAFs; green, downregulated DAFs. DAF, differentially accumulated flavonoids; KEGG, Kyoto Encyclopedia of Genes and Genomes.

The metabolome analysis of the samples revealed a total of 328 flavonoids, making up 99.39% of all detected metabolites (**Table S5**). The correlation coefficient of variation of >85% of the samples lay below 0.5, and the correlation between groups ranged between 0.6 and 1.0 (**Table S6**). These data indicated that the metabolome data quality was reliable and suitable for further analysis. DAFs of the metabolomes of Jimai229 and HHG46 with vs without exposure to powdery mildew were identified. In the JM229-pm/JM229-0 set, 129 metabolites were significantly upregulated and 21 were downregulated; in the HHG46-pm/HHG46-0 set, 131 were upregulated and 13 were downregulated (**Figures 2A, B**). The most commonly enriched KEGG terms for the HHG46-pm/HHG46-0 DAF dataset were ‘flavone and flavonol biosynthesis’ (76.92%), ‘flavonoids biosynthesis’ (30.77%), ‘biosynthesis of secondary metabolites’ (30.77%), ‘metabolic pathways’, and ‘anthocyanin biosynthesis’. ‘Flavone and flavonol biosynthesis’ was also the most enriched KEGG pathway in the JM229-pm/JM229-0 data at 68.42%, indicating that the flavone and flavonol biosynthesis pathway was the most activated pathway under conditions of powdery mildew treatment (**Figures 2C, D**). The top 20 DAFs in Jimai229 and HHG46 with vs without inoculation suggest that flavones and flavonols were the metabolites that were most heavily accumulated in response to powdery mildew infection (**Figures 2E, F**). Overall, the data strongly suggested that the flavone and flavonol biosynthesis pathway serves a role in powdery mildew resistance in wheat.

### Profiles of DEGs and DAFs in flavonoids biosynthetic pathways associated with powdery mildew resistance

**Figure 3:**
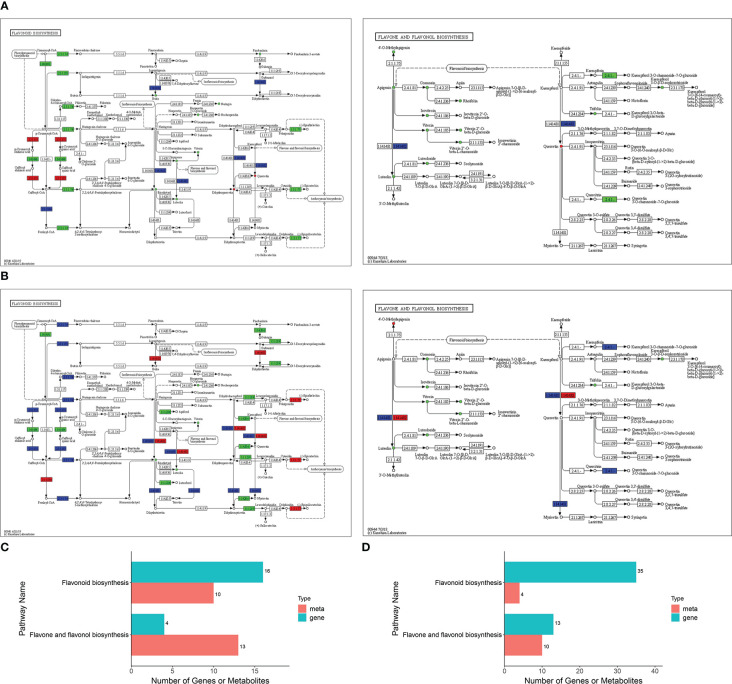
Integrated analyses of the identified differentially expressed genes and differentially accumulated flavonoids. KEGG enriched pathways for **(A)** JM229-pm/JM229-0 and **(B)** HHG46-pm/HHG46-0. Number of identified genes and metabolites involved in these KEGG pathways for **(C)** JM229-pm/JM229-0 and **(D)** HHG46-pm/HHG46-0. KEGG, Kyoto Encyclopedia of Genes and Genomes; meta, metabolite.

The transcriptome and metabolome data obtained in the present study were found to be highly correlated (**Table S7**). Two pathways were found to be highly enriched in both Jimai229 and HHG46 following inoculation with powdery mildew: ‘flavonoids biosynthesis’ (P=0.009 for JM229-pm/JM229-0; P=0.0004 for HHG46-pm/HHG46-0) and ‘flavone and flavonol biosynthesis’ (P=0.3422 for JM229-pm/JM229-0; P=0.0062 for HHG46-pm/HHG46-0) (**Figures 3A, B**; **Table S8**). To better understand the relationship between the identified genes and metabolites, the DEGs and DAFs resulting from the above analyses were simultaneously mapped to the KEGG pathway diagram. For cv. Jimai229, a total of 16 key genes and 10 metabolites were simultaneously mapped to the flavonoids biosynthesis pathway (ko00941), and 4 genes and 13 metabolites to the flavone and flavonol biosynthesis pathway (ko00944). For HHG46, 4 key metabolites and 35 genes were simultaneously mapped to flavonoids biosynthesis, and 10 metabolites and 13 genes to flavone and flavonol biosynthesis (**Table S8**; **Figures 3C, D**). These results further reinforced the key function of flavones and flavonols in the resistance of wheat to powdery mildew.

### Confirmation of flavonoids synthesis gene expression

**Figure 4:**
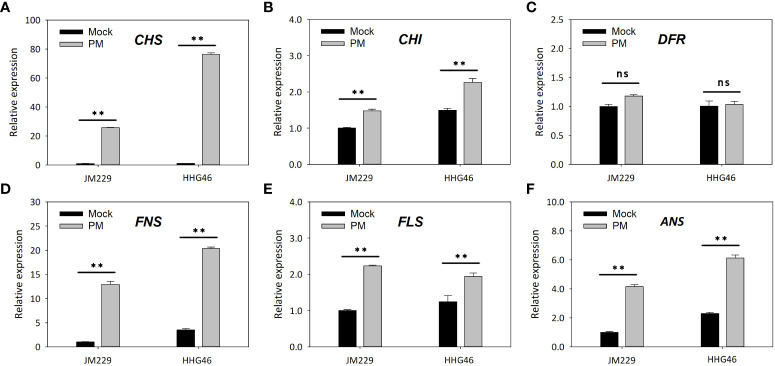
The effect of powdery mildew on the expression level of flavonoids synthesis-related genes by quantitative reverse-transcription PCR analysis. Relative expression levels of **(A)** *CHS*, **(B)** *CHI*, **(C)** *DFR*, **(D)** *FNS*, **(E)** *FLS*, and **(F)** *ANS* in susceptible wheat cv. JM229 and resistant cv. HHG46. Error bars represent mean standard deviation of triplicate experiments. **, mean values differ significantly at the 1% probability level. ANS, anthocyanidin synthase; CHI, chalcone isomerase; CHS, chalcone synthase; DFR, dihydroflavonol 4-reductase; FLS, flavonol synthase; FNS, flavone synthase; ns, not significantly different; PM, powdery mildew-inoculated group.

qRT-PCR showed that, compared to the mock-treatment group, powdery mildew infection led to increased expression levels of the *CHS*, *CHI*, *FNS*, *FLS*, and *ANS* genes in both Jimai229 and HHG46, while no significant difference was observed in the relative expression of *DFR* (**Figure 4**). These results confirmed that the flavonoids synthesis pathway was upregulated following powdery mildew infection, and indicated that these genes were expressed more strongly in the resistant cv. HHG46 than in the sensitive cv. Jimai229, under both mock and powdery mildew treatment conditions. These findings corroborated the reliability of the RNA-seq results of this study and further supported the hypothesis that flavonoids play a key role in powdery mildew resistance in wheat.

### Flavonoids accumulation induced by powdery mildew infection

**Figure 5:**
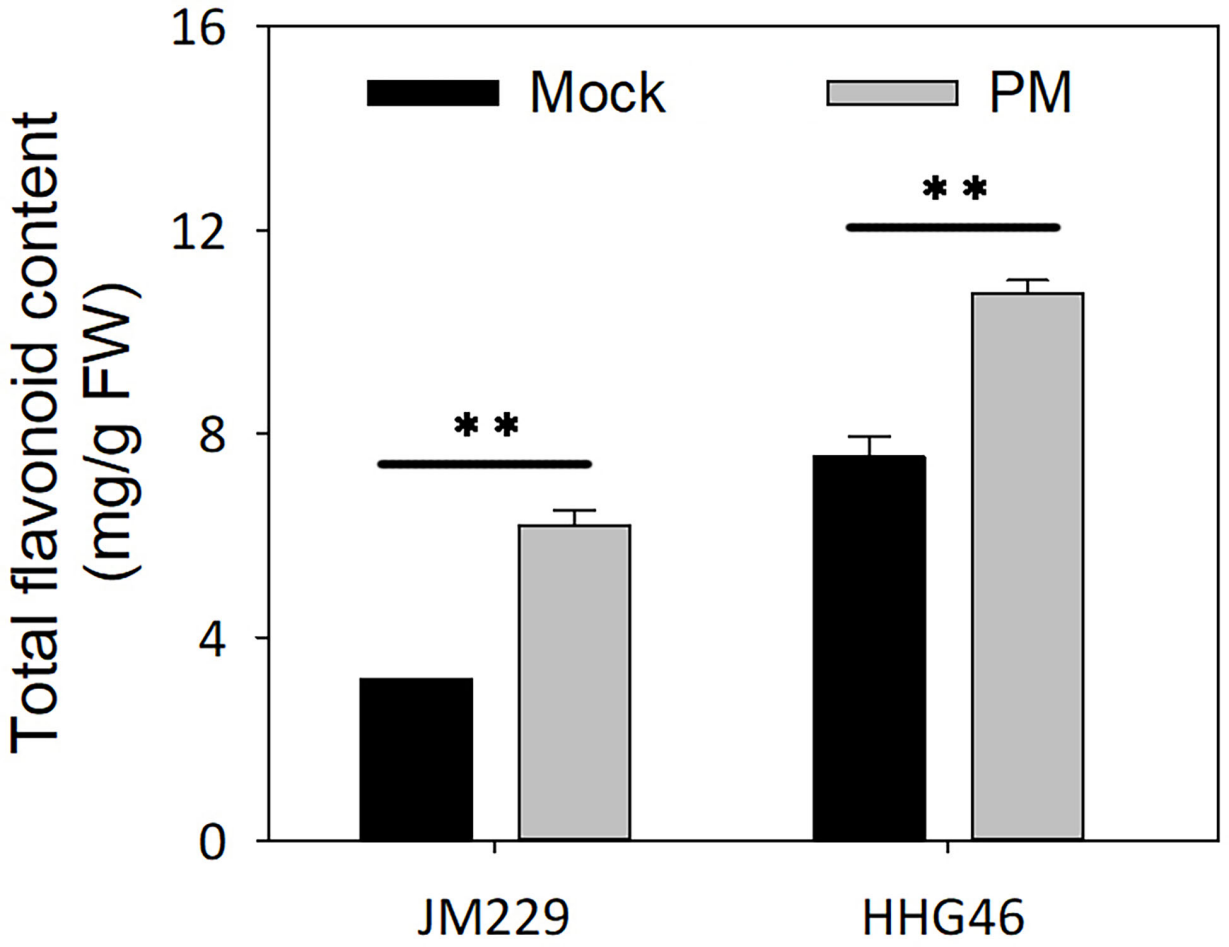
Total flavonoids content of wheat cv. Jimai229 and HHG46 under mock and PM conditions. Error bars represent mean standard deviation of triplicate experiments. **, mean values differ significantly at the 1% probability level. FW, fresh weight; PM, powdery mildew-inoculated group.

Powdery mildew inoculation was found to lead to a 2-fold and 1.4-fold increase in total flavonoids content in cv. Jimai229 and HHG46, respectively, compared to the mock-treatment group. Furthermore, the total flavonoids content in the HHG46 plants was 2.4 times higher than that in the Jimai229 plants under control conditions, and 1.73 times higher under conditions of powdery mildew treatment (**Figure 5**). These results suggested that powdery mildew infection leads to the stimulation of flavonoids synthesis in both Jimai229 and HHG46, and that this may contribute to the resistance of HHG46. Overall, these data were in agreement with the metabolome analysis results.

### Exogenous flavonoids treatment of powdery mildew-infected wheat

**Figure 6:**
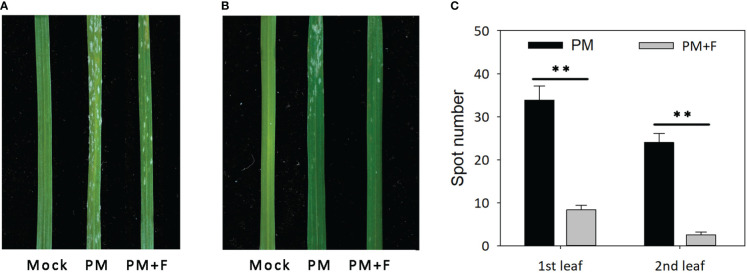
Effects of powdery mildew and flavonoids treatment on leaf morphology in cv. Jimai229. Images of the representative phenotypes of the **(A)** first leaves and **(B)** second leaves from the mock, PM, and PM+F groups. **(C)** Plot of the number of white spots observed on the leaves under the different conditions. Error bars represent mean standard deviation of triplicate experiments, with 10 leaves included per replicate. **, mean values differ significantly at the 1% probability level. PM, inoculated with powdery mildew; PM+F, inoculated with powdery mildew and treated with 100 μM flavonoids solution.

The development of powdery mildew spots was observed on the leaves of cv. Jimai229 plants with and without treatment with exogenous flavonoids solution (**Figure 6**). Compared with the mock-treatment group (no powdery mildew), the first leaves of the inoculated plants were severely infected, presenting with a large number of white spots and beginning to curl. The second leaves developed a lower number of white spots in the apex compared with the first leaves. The PM+F group exhibited significantly fewer white spots, with a 75.4 and 89.6% reduction on the first and the second leaves, respectively, compared with the inoculated plants without flavonoids treatment (PM group). Overall, the Jimai229 plants in the PM group were classified as 4, and the PM+F group as 2. Therefore, flavonoids appear to function in the resistance of wheat to powdery mildew.

### Role of flavonoids in powdery mildew resistance

**Figure 7:**
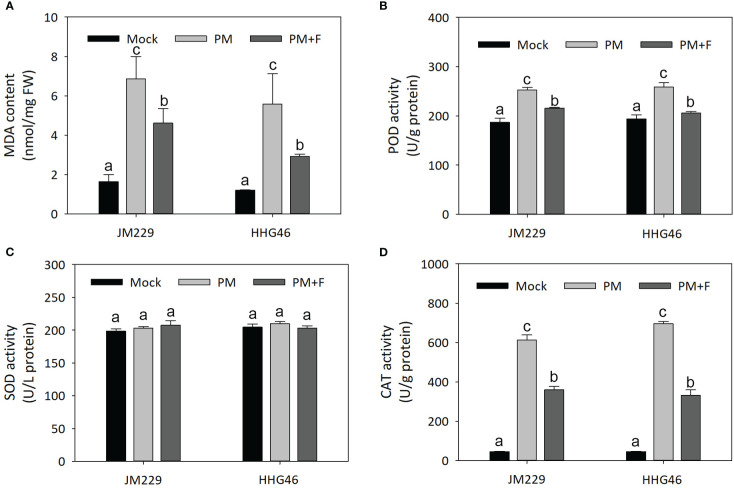
Defense-related responses against powdery mildew with or without flavonoids treatment in wheat leaves. **(A)** MDA content. Activity of **(B)** POD, **(C)** SOD, and **(D)** CAT in cv. Jimai229 and cv. HHG46 under mock, PM, and PM+F treatment conditions. Error bars represent mean standard deviation of triplicate experiments. Different letters represent significant differences at the 5% probability level. CAT, catalase; MDA, malondialdehyde; PM, inoculated with powdery mildew; PM+F, inoculated with powdery mildew and treated with 100 μM flavonoids solution; POD, peroxidase; SOD, superoxide dismutase.

The MDA content, as well as the POD, SOD, and CAT activity were measured in the plants of the mock, PM, and PM+F groups, revealing similar trends for the two wheat cultivars (**Figure 7**). In comparison to that in the mock-treatment group, the MDA content in the plants inoculated with powdery mildew was significantly higher by a factor of 3–4, and POD and CAT exhibited higher activity. On the other hand, the plants in the PM+F group displayed an almost two-fold lower MDA content, a slight reduction in POD activity, and a marked decrease (~2x) in CAT activity, compared with the PM group. No significant differences were observed in the activity of SOD among the different treatment groups. These results suggested that, upon powdery mildew infection, CAT was activated to scavenge any resulting reactive oxygen species (ROS). The addition of exogenous flavonoids appeared to reduce the harm caused by powdery mildew to the wheat plants, but also seemed to have inhibited the ROS scavenging system. Overall, flavonoids may play a dual role in powdery mildew resistance: on one hand, they may lessen the adverse impact of the disease by reducing POD activity and MDA content; on the other hand, they may hinder the ROS scavenging activity and maintain ROS homeostasis in wheat.

## Discussion

### The potential of flavonoids for biological control against powdery mildew in wheat

Flavonoids comprise one of the most crucial and diverse phenylpropanoid groups in plants (Dong & Lin, 2021). These metabolites cause a wide variety of colors in plants, scavenge ROS, and are used for curing human diseases (Iwashina, 2003; Grotewold, 2006; Pourcel et al., 2007; Cavaiuolo et al., 2013; Fernandes et al., 2017; Zhang et al., 2020; Selvakumar et al., 2020). Previous studies have reported that flavonoids confer resistance to powdery mildew in strawberry and cucumber (Fofana et al., 2005; Bajpai et al., 2019); however, the function of flavonoids in such resistance in wheat has not been investigated to the best of our knowledge. Herein, KEGG analysis of DEGs identified following inoculation of wheat plants with powdery mildew revealed that flavonoids synthesis pathways were enriched, and the upregulation of such genes was confirmed by qRT-PCR. Flavonoids-targeted metabolome data indicated that the flavone and flavonol synthesis pathway was the most enriched pathway responding to powdery mildew infection, and total flavonoids content was also found to be increased. The combined transcriptome and metabolome findings suggest that flavonoids are most likely involved in powdery mildew resistance in wheat.

### Integrated transcriptome and metabolome analyses

Multiple-omics technologies have emerged as powerful tools to study plant systems, including data from genomics, transcriptomics, proteomics, metabolomics, and ionomics, among others. Transcriptomics cover the transcriptome, which refers to the complete set of RNA transcripts produced by an organism or tissue (Raza et al., 2021). Transcriptome profiling is dynamic and is proving to be a promising technology for the analysis of gene expression in response to any stimuli over a given time period (Yang et al., 2021). Metabolomics is defined as the comprehensive study of metabolites that participate in different cellular events in a biological system (Yang et al., 2021). Combined analyses of transcriptome and metabolome data have led to the identification of the molecular, hormonal, and metabolic mechanisms associated with powdery mildew infection in grapes, and have helped develop metabolic biomarkers such as gallic and eicosanoic acid in the early stages of infection (Pimentel et al., 2021). Transcriptome sequencing has revealed the positive effect of *B. subtilis* on controlling powdery mildew in wheat, by stimulating the salicylic acid-dependent signaling pathway, resulting in the discovery that these bacteria can be used as biocontrol agents against fungal disease (Xie et al., 2021). In the present study, transcriptome analysis and flavonoids-targeted metabolome analysis were used to identify genes and flavonoids involved in powdery mildew resistance in wheat. Combining the data from the transcriptome and metabolome analyses revealed that the flavonoids synthesis pathway and the corresponding metabolites were enriched following powdery mildew inoculation. The most enriched DAFs were found to be flavones and flavonols.

### Flavonoids may have dual function in wheat resistance to powdery mildew

When plants are challenged by a disease, their redox system is activated to combat this threat. MDA content and POD activity are commonly used to evaluate the degree of damage caused by disease. The production of MDA causes oxidative damage to proteins and nucleotides and aggravates oxidation of the cellular lipid membrane (Mohapatra et al., 2016); POD production usually occurs in aging organs (Dong et al., 2017). In the process of fighting oxidative damage, the activity of antioxidant enzymes is induced. SOD clears the damaging free radicals by catalyzing the conversion of the superoxide anion to oxygen and water (De Pace et al., 1988; Lightfoot et al., 2017). CAT, another important redox marker, reduces hydrogen peroxide to oxygen and water (Gao et al., 2021). ROS are essential for any well-functioning organism, as they activate the immune response through increasing the release of inflammatory factors. They also act as signaling molecules that regulate and maintain normal physiological functions (Mittler, 2017; Hu et al., 2020). It is therefore critical for healthy plants to maintain a balanced ROS concentration.

The function of flavonoids in the resistance to powdery mildew has been investigated for many years. The study of DAMs and DEGs of susceptible and resistant cucumber strains revealed that the flavonoids synthesis pathway and flavonoids-related metabolites were involved in powdery mildew resistance (Zhang et al., 2021). Additionally, transgenic expression of *VqWRKY31* in *Vitis vinifera* conferred resistance to powdery mildew by activating salicylic acid signaling and flavonoids synthesis (Yin et al., 2022). However, the mechanism of flavonoids aiding resistance to powdery mildew infection in wheat has not been elucidated. Our results suggested that flavonoids conferred resistance to powdery mildew in wheat by regulating the redox system: on one hand, they may reduce the adverse effects of powdery mildew by decreasing POD activity and MDA content; on the other hand, they may decrease CAT activity to maintain a balance of ROS and homeostasis to support the defense-related signaling cascade.

## Data availability statement

The raw data supporting the conclusions of this article will be made available by the authors, without undue reservation.

## Author contributions

CL and WX designed the experiments. WX, XX, RH, XW, and KW performed the experiments. WX and GQ analyzed the data. WX, PM, CL, and TK wrote the manuscript. All authors contributed to the article and approved the submitted version.
